# Energy Efficiency in Public Buildings through Context-Aware Social Computing

**DOI:** 10.3390/s17040826

**Published:** 2017-04-11

**Authors:** Óscar García, Ricardo S. Alonso, Javier Prieto, Juan M. Corchado

**Affiliations:** BISITE Research Group, Edificio I+D+I, University of Salamanca, 37007 Salamanca, Spain; ralorin@usal.es (R.S.A.); javierp@usal.es (J.P.); corchado@usal.es (J.M.C.)

**Keywords:** behavioral change, serious games, context-awareness, collaborative learning, energy efficiency, social computing, virtual organizations

## Abstract

The challenge of promoting behavioral changes in users that leads to energy savings in public buildings has become a complex task requiring the involvement of multiple technologies. Wireless sensor networks have a great potential for the development of tools, such as serious games, that encourage acquiring good energy and healthy habits among users in the workplace. This paper presents the development of a serious game using CAFCLA, a framework that allows for integrating multiple technologies, which provide both context-awareness and social computing. Game development has shown that the data provided by sensor networks encourage users to reduce energy consumption in their workplace and that social interactions and competitiveness allow for accelerating the achievement of good results and behavioral changes that favor energy savings.

## 1. Introduction

In the last several years, energy efficiency has become a capital policy for many countries around the world. It is widely recognized as the most cost-effective and readily available means of addressing numerous energy-related issues, including energy security, the social and economic impacts of high energy prices, and concerns about climate change [1]. As a consequence, there have been many efforts to develop and deploy hardware solutions (smart meters, smart plugs, smart sensors, etc.) and sophisticated control structures to measure and control energy consumption in grids, buildings and households, and these solutions have been proved to be technologically affordable and sustainable [2,3].

One of the most important problems to solve, both technically and socially, is energy consumption in public spaces and work environments. Within these environments, the building sector plays a crucial role as far as energy consumption is concerned, thus efforts and resources are used in making them more efficient [4]. Energy waste in these areas is quite high due to several reasons. On the one hand, the implementation of technological solutions that enable efficient energy use is not spread out among old buildings or, at least, among those above a certain age. On the other hand, users’ comfort level is very difficult to model because of the disparity of requirements among each user, making it difficult to deploy automatic energy management systems in buildings [5]. However, above these and other reasons, the most important issue that affects the misuse of energy in public buildings is the users’ behaviour, which generally wastes these resources carelessly (do not turn off lights, leave monitors or PC working all night, make needless use of lifts, etc.), largely because they have no direct awareness of the generated energy bill [6].

Sensor networks have been postulated, by their own nature, as an optimal technology for the fulfilment of efficient energy use in public buildings [7]: some of the proposed solutions use these networks to determine what enhancements are needed when improving the air conditioning in building climate [8]; other systems use presence sensors and timers to manage lighting; techniques based on the analysis of consumption of devices through smart meters are commonly used to give feedback and educate users [9]; finally, other works go further and develop intelligent control systems to act on resources, such as the HVAC (Heating, Ventilation and Air Conditioning) [9], or consider the integration of automation platforms [10]. However, due to numerous reasons, the results are far from expected: modelling, data analysis or automation are sometimes inefficient, intelligent systems do not usually take into account users’ requirements and, the most important issue, not all of them influence a sustained change in users’ behaviour.

Most of the abovementioned methods require users’ wilfulness to analyse information and change their habits in order to reduce consumption [3]. To improve this aspect, it is necessary to find mechanisms that encourage and educate users on the efficient use of energy, produce greater energy savings through user loyalty [11]. In this sense, serious games have great potential because they are designed to attractively educate and promote changes in the behaviour of its participants [3,12]. Users enjoy, have fun and interact among them while gaining awareness of the problem to be solved [13]. Offering tangible incentives enables more effective learning of the habits sought. Furthermore, the development of these games over extended periods of time can reinforce the objectives achieved, so that users acquire behavioural changes gradually and transparently over time [13]. Until now, several serious games have been developed in combination with sensor networks such as IBM CityOne Game, which proposes real problems related to the environment and energy saving that users have to solve [13]; EnergyLife, which provides awareness of the consumption of electrical devices through sensor networks and awards users with points if this awareness improves with time [14]; Power Explorer, which also works with data from sensors to raise young people’s awareness with regards to energy consumption [15]; or Energy Chickens, where the health of a virtual chicken is reflected by the user’s energy savings when using some selected devices [3].

The development of serious games imposes an interesting challenge since they have to succeed in improving motivation, encouraging participation and enhancing the learning process. To address this challenge, Context-aware Learning is presented as an alternative with high interest because: by collecting real time data, it obtains the characteristics of the environment in which the game is taking place; it provides knowledge on the position and status of both objects and people; and, more importantly, it enables the customization of the game content depending on the needs of each user and their surrounding environment [16]. Context-aware Learning systems use the information gathered from the environment to enhance users’ learning and behavioural change [17]. Context-aware systems make use of Wireless Sensor Networks (WSNs) and Real Time Locating Systems (RTLS) to collect information that enables monitoring, replicating or interacting with the environment where the game is taking place [18]. WSN and RTLS provide dynamic, efficient and flexible infrastructures that collect contextual data regardless of the chosen location [19].

Despite all the examples mentioned above, the combination of sensor networks with serious games has not obtained satisfactory results so far. Firstly, they have not reached high levels of loyalty, and, secondly, results that would prove a substantial change in the behaviour of participants have not been achieved yet. It is therefore necessary to provide custom tools that take into account the habits, preferences, ambitions and interactions of people that can be sustained over time. In this paper, the implementation of social computing by means of virtual organizations of agents is proposed as the best alternative to cover all of these needs. This approach requires the use of mechanisms that connect multiple devices and manage the information collected intelligently [20]; it allows a more efficient management of the information collected by hardware resources; it enables higher levels of personalization in the services provided to users that derive from sustained and efficient actions over time; it facilitates the administration and development of the game; and it provides the necessary background to support the design, development and deployment of systems in which the communication and management of WSNs require advanced competences [20].

The aim of the work presented in this paper is to provide a new framework for the development of context-aware serious games that spark a behavioural change towards more energy efficient habits. The development of the framework is carried out using CAFCLA (Context-Aware Framework for Collaborative Learning Applications) as a basis for its technical and social features implementation [21]. The social computing perspective has been taken into account when designing the framework, permitting the use of social and contextual information. The contextual information required is gathered through the deployment of a WSN that facilitates the acquisition of data related to the energy consumption from different aspects (temperature, luminosity), the presence of users in certain places or the efficient use of electronic devices and HVAC systems. In addition, the RTLS permits determining what behavioural habits users follow and gives guidelines on how to improve these habits in their work and transits. A virtual organization of agents supports the social machine, and this gives intelligence to the game and enables and enhances the learning process [22,23], updating contextual information, monitoring users’ actions, providing information to players or facilitating the deployment, configuration and communication of the WSN and RTLS deployed. The framework has been assessed through an experimental implementation of a serious game, which intends to improve users’ habits with regards to efficient energy use in a public building. The main innovations presented here are:

- the use of WSN and RTLS in a public building, to deploy an individual and at the same time, collective game between users;
- the management of this game through the CAFCLA framework and a virtual organization of agents, to improve game development and deployment, and the integration of technologies; and;
- the use of these technologies within the paradigm of social computing, in order to enable higher customization and collective interaction.

The rest of the paper is structured as follows: Section 2 describes the context surrounding the work presented, including research efforts to: motivate behavioural change of consumers, design serious games for energy efficiency, apply context-awareness for energy savings in buildings, propose context-aware learning tools, and develop social computing machines to deploy this sort of games. Section 3 depicts the deployed system that creates the serious game, including a comprehensive description of both the framework and each of the components conveying the different layers. Section 4 details the experiment and discusses the results obtained through an empirical implementation in a real scenario. Finally, Section 5 exposes the main conclusions that have emerged from the research and experimentation.

## 2. Previous Works

The work presented hereafter inquires into the process of obtaining a more efficient use of energy and the users’ behavioural change necessary to obtain it. To accomplish this issue, the section we present addresses previous works on this topic. Furthermore, in the analysis of the state-of-the-art, different solutions that make use of serious games are analysed. Context-aware and energy saving in buildings are depicted in order to identify solutions and tendencies within this field. More specifically, our work emphasizes the benefits of context-aware learning, an effective way of changing user behaviour, paying particular attention to the sensor infrastructure that has to be deployed in order to obtain an operative framework. Finally, any system must be properly managed so, to this end, social computing and virtual organizations of agents are presented as an alternative that meets all of the requirements sought by our system.

### 2.1. Behavioural Change for Energy Efficiency

There are many studies that support the theory of technical implementations being more effective if accompanied by a change in the users’ behaviour, and there are even trends that give more value to the change of consumption practices than to technical implementation [24]. Therefore, it is necessary to model the behaviour of users to determine what the users do, where they do it and why they do it [25]. The design of models that promote behavioural change differs, depending on whether it is for domestic or non-domestic users. While home users have a direct contact with energy costs, the policies implemented in the non-domestic sector are made at the organizational level. Consequently, they lack a direct relationship with the behaviour, habits and benefits for the users, workers, or other stakeholders. For this reason, motivating such users to acquire good energy saving habits is an arduous task that requires a firm commitment on the part of the corporations and has to promote the use of attractive tools that support and reinforce the acquisition of these habits [26].

The direct relationship between the users and the expenses related to energy consumption in their homes has made this area the focus of research [27]. Among the proposed solutions within households are those based on providing feedback to users on their consumption by using advanced metering infrastructures, such as smart meters [28], displays reporting real-time consumption [29] or even recommendations on the use of more efficient appliances [30]. However, the high penetration of Information and Communication Technologies (ICT) in the society has facilitated the development of other solutions that take into account useful social information for users to encourage behavioural change among users [31]. These solutions include disciplines such as media, entertainment or gaming [12].

Despite these efforts, research and proposed solutions to promote behavioural change in public buildings and offices have not yet been deeply addressed [32], although many studies show that it is feasible to reduce energy demand in office by changing user habits, behaviours and lifestyle [33,34]. More specifically, real-time monitoring of energy consumption, feedback to users and behavioural change can affect up to 40% of the whole energy consumption in a public building [34]. Some of the proposals that have been made so far have extrapolated the solutions used in homes to offices, such as the use of smart meters [33] or the efficient management of HVAC systems [34]. However, promoting behavioural change in users requires that they are aware of the benefits of an efficient energy use [35], as well as the efficient use of the most demanded resources, such as MELs (Miscellaneous Electric Loads, including PC, scanners, printers, etc.) [36], which consume more than 25% of energy in offices [36]. Much of the strategies that resolve this issue are based on providing awareness among users in different ways: through interventions that promote their sustainable use [37], through interactive posters and motivation of users [38] or by giving feedback via email or other communication channels [36].

The work described in this paper aims to cover existing gaps in these incentive methods through a more attractive use of the technology. Concretely, it develops games that help users to learn good habits that favour saving energy at the office. The following subsection deals with the use of serious games as a tool for behavioural change that promotes energy efficiency.

### 2.2. Serious Games

As it is widely recognized, games are usually related to entertainment and, although they have always been applied in the educational processes, the growth and the implantation of the ICT have allowed them to become more popular. Serious games have a specific purpose related to learning, understanding or social impact, addressing both cognitive and affective dimensions by the application of game design, dynamics and concepts that stimulate and make more attractive the interaction of the student with the learning process.

Serious games are a broad trend in which traditional mechanisms of games are used in multiple environments such as public policies [39,40], defense [41,42], business management [43,44], healthcare [45,46], education [47,48] or energy saving [49,50,51], among others [52]. The main objective of these games is to teach, paying special attention to the educational purpose beyond entertainment, but not neglecting it. In this way, games allow users to acquire skills through play-based activities by using their inherent playfulness and interactive characteristics. They also facilitate the motivation, training and engagement of participants, as well as their learning process by improving the performance of a specific objective through the acquisition of new knowledge and skills [53,54].

Serious games have the optimal characteristics that can influence user behaviour to be more energy efficient in public or working environments [43]. They have been used in different areas to promote this change [55,56]. One of the main reasons for their use is that games allow higher levels of engagement and stimulate innovation, and the use of short cycles feedback favours this change, as well as making well-defined rules or defining achievable short-term goals.

Various approaches to serious games in the energy sector are being piloted or commercially deployed, each adopting differing gamification techniques and having different key objectives [3,49,50,51]. In [3], the authors develop a serious game to save energy and change behaviour in an office environment, saving 13% in energy consumption, but the behaviour change does not persist in time. In [12], the researchers present a social game to promote energy saving behaviour by giving information to the consumers through a game, but results show a small drop in the use of energy (around 2%). A common factor is their use of granular and real-time energy data, which allows them to provide instantaneous feedback. Moreover, most of these solutions are based on the awareness given to users in the attempt of raising and enhancing this awareness. However, the implementation of serious games in office environments is hardly used and is mostly based on providing awareness of energy consumption and promoting energy saving [3].

Despite the multiple solutions and researches that have been analysed, the exploitation of ICT within this field can be performed. One of the main weaknesses found is related to the use of WSNs when designing and deploying serious games for energy efficiency. While the technology used is practically based on obtaining consumption, mostly from smart meters, the WSNs offer a great potential to collect other kinds of environmental data that could create richer activities from the parameters on user behaviour obtained during the game. The following subsections outline the importance of context-awareness and energy saving in buildings, and, after that, the contextual information in the learning process focusing on its benefits.

### 2.3. Context-Aware and Energy Saving in Buildings

As mentioned in previous sections, real-time monitoring of energy consumption, among other factors, can affect up to 40% of consumption in buildings [34]. In this sense, context-awareness systems become the ideal technology to obtain real-time environmental information from the locations to be characterized and where energy savings are encouraged [10].

Throughout the literature, multiple works have been presented in order to optimize energy efficiency in buildings by using context-aware technologies [32,33,34,57,58,59,60,61]. The design and deployment of context-aware systems allow for determining which factors influence the energy consumption at any time, including the use of devices (open window, light on, open blinds, etc.) [32,33,34,57], environmental conditions (temperature, humidity, lighting, etc.) [57,58,59] or the users’ location (home, work, market, etc.) [60,61].

Among the works focused on providing contextual information to facilitate energy savings, Han et al. propose a semantic service that allows for integrating multiple sensors [58]. Their main objective is to favour the automation of tasks in buildings and thus improve the energy consumption. Kamienski et al. recognize the difficulty of integrating contextual technologies in real environments [59]. To solve this problem, they have designed IMPReSS, a project based on the rapid integration of IoT (Internet of the Things) technologies but that is still in the implementation phase. Kamilaris et al. considers that monitoring of electricity consumption is highly important in buildings. They combine it with the integration of environmental sensors, device profiles and occupancy information in order to develop a richer source of information and more potential data to be used [34].

Moreover, there are other works in which a more precise and concrete contextualization is done. Thomas et al. integrate sensors to measure luminosity, temperature, doors and blinds status, etc., as well as a location system through movement sensors [57]. The system presented facilitates energy saving thanks to the execution of rules based on registered behaviours and activity awareness. Other studies present occupancy identification and locations systems as the newest trends in this kind of solutions. Indoor location via RFID+IR (Radio Frequency Identification + InfraRed) systems [60] or those based in BLE (Bluetooth Low Energy) [61], improve energy efficiency by automating some tasks or by customizing services [61].

Despite of all the above, there is a lack of solutions that address the problem from a global point of view and that offer flexible and adaptable solutions to a large number of use cases, abstracting the underlying technology from problem solving, as it has been developed in this paper.

### 2.4. Context-Aware Learning

Context-aware Learning arises from the inclusion of context-awareness in the learning process [62,63]. Thus, the educational process takes advantage of the flexibility provided by the use of real-time environmental information within the process [64]. Moreover, the use of technologies that allow for obtaining contextual information, such as WSN or RTLS, enriches the learning process [64].

Serious games benefit from the inclusion of contextual and location data for their design and development [65,66]. From this point of view, sensor networks make it easier to obtain an accurate “energy picture” of the environment in which the game is played [8,9,10]. At the same time, sensors allow for acting automatically on the environment once the parameters of user behaviour are determined. In addition, the ability to locate users in real time allows for launching challenges that promote and enhance both energy saving and the acquisition of good habits. In [8], sensors are used to acquire data that permits modeling a building in terms of energy consumption and use this information to improve its use. In [9], temperature and humidity sensors collect data to improve the use of the HVAC system, achieving a more comfortable working or home environment. In [10], a system helps designers to select the best parameters to control energy consumption, getting up to 23% energy savings in a real scenario.

Many games developed for encouraging efficient energy usage do not consider the use of sensor networks and real-time location of users to enrich the learning process. Similarly, it is unusual to find solutions designed for the workplace, so the immersion of wireless sensor networks in these environments to promote good energy habits seems very productive. The solutions in the aforementioned research integrate different technologies to meet the objectives of a specific game. Moreover, there is no generalization in the proposals, so they cannot be used for any purpose other than the one defined by their authors. Finally, the use of intelligent management techniques is not taken into account in the researches; these techniques improve the game and the behavioural change through prediction, adaptation and anticipation of users’ actions.

The next subsection introduces the social computing paradigm and justifies the added value that it offers to the process of behavior change sought in the research described in this paper.

### 2.5. Social Computing

Recent tendencies have led to the social computing paradigm for designing social systems that helps us build sociotechnical tools where humans and machines collaborate to resolve social problems [67,68]. These tools have a high level of complexity and require the use of artificial intelligence to manage artificial societies; however, they have the capacities needed to provide effective collaboration between humans and machines [68]. Virtual organizations of agents are particularly well suited as support for the development of these systems [69]. They enable the description of structural compositions and functional behaviour, and the inclusion of normative regulations for controlling agent behaviour, for the dynamic entry/exit of components and for the dynamic formation of agent groups [70].

The social computing paradigm aims to create social entities managed both by technology and social processes known as Social Machines. These entities allow systems to generate recommendations to users, such as the social machine developed by Amazon [71], to perform a task through the collaboration of a human and a machine, as it is found in the CAPTCHA (Completely Automated Public Turing test to tell Computers and Humans Apart) system to authenticate users [72], or to predict social dynamics from behaviour data, as it is done by Twitter [73].

At the same time, social networks have become one of the most used Internet activities. Opinions on products, services, etc. posted in social networks increasingly impact the decision making process for purchases or even choosing a service. It is also known that social networks enhance engagement in activities or games. Currently, there are many initiatives to engage people in behavioural change using known social networks such as Facebook or Twitter. There is a number of proposals that address the problem of behavioural change for energy efficiency by using serious games through social networks [38,40]. However, human–machine interactions are not deeply addressed in these solutions leaving aside—for example, contextual information that may be useful in the field of our focus. Moreover, these proposals do not offer working infrastructures that allow for integrating different technologies, various communication protocols, diverse ways of promoting social relations, or the intelligence for managing the system based on the needs of the game.

In the remainder of this paper, a framework is proposed based on social computing and context-awareness that enables us to create learning scenarios, such as serious games, which encourage and enhance a change of behavior in consumers so that they use energy more efficiently in their workplace.

## 3. System Overview

This section addresses all the issues related to the deployment of a serious game in a way that encourages users to use resources efficiently and manages to change their habits in an office environment. The work depicted here uses the framework proposed previously CAFCLA (Context-Aware Framework for Collaborative Learning Activities) [21], focused on collaborative learning through the use of contextual information.

### 3.1. Background

As mentioned above, CAFCLA is a framework whose main purpose is the integration of different technological resources to make the design and development of learning activities easier, based on contextual information and social computing. Within this context, CAFCLA allows its users to have multiple resources offered so that, through them, the use of contextual information and social interactions is simplified. In addition, this integration does not only help to design learning activities, but also enables a faster start by reducing the time taken for its development.

CAFCLA has been used for collaborative learning activities that use contextual information in museums [74], gardens [21] and other educational settings [64]. In this paper, CAFCLA is used in a non-academic environment with a specific purpose: to educate, raise awareness and trigger behavioural change in the efficient use of energy in public buildings. To this end, we have designed and developed a serious game whose function is to make users aware, acquiring good habits naturally and changing their behaviour, so that they save energy by using it more efficiently.

In general, the system continuously monitors the use of lighting, the use of HVAC systems, the electrical energy consumption at the site of each user, the temperature and luminosity of the environment, and the location of users through a WSN. Thus, all data are obtained in real time according to the activity in the laboratory, its temperature, if the use of lighting is being efficient, if users turn off or suspend their devices when they leave their job, and whether users use an elevator or the stairs to reach the lab. All of these data will enable us to check if users meet certain energy efficiency targets or collective challenges. If so, users will be rewarded with virtual coins and penalized otherwise.

In this game, the collection of data through sensors, the location of participants and the social interactions among them and with the environment are of special relevance. CAFCLA provides the tools required to efficiently manage the game and the interactions generated. All of these aspects are discussed in depth in the next section where we describe in detail each component of the framework and what they are used for in this case.

### 3.2. Framework Description

The game designed using CAFCLA requires the integration of different physical devices and technologies. As shown in Table 1, CAFCLA has been designed following a scheme of interconnected layers. Each layer includes a set of technologies that fulfil the requirements of the game. These devices and technologies will support communication, sensor data collection and contextualization of the environment, and provide intelligence to the system and even facilitate the development of the application used by players.

In the following sections, and with the objective of understanding the functionalities of CAFCLA better, a description of each layer is provided. A brief explanation is also included on the reason for each technology selected and its function.

#### 3.2.1. Physical Layer

**Figure 1:**
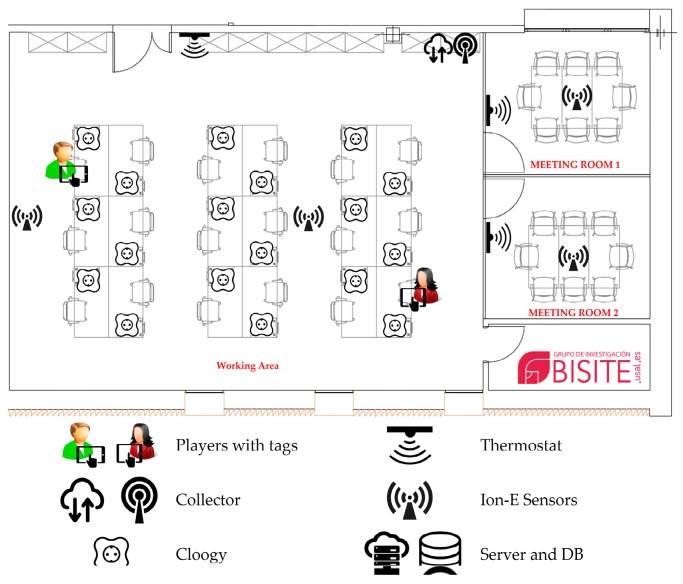
The CAFCLA (Context-Aware Framework for Collaborative Learning Applications) framework enables the integration of heterogeneous sensors as well as communication technologies in the physical layer.

The physical layer contains all of the devices that will be used in the framework (see Figure 1):

- An infrastructure that collects all contextual information: temperature on/off, luminosity and consumption sensors, integrated into plugs that monitor the power consumption of each job site.
- Location beacons and identification tags that obtain the position of users.
- Mobile devices to deploy, modify and access the game: tablets, laptops and smartphones.
- Internet access points via Wi-Fi and Ethernet, as well as data collectors and hubs, to send the data collected by sensors as well as by the real-time locating system.
- A server to store data and run the application.

All of these technologies are integrated by CAFCLA transparently to users, making an appropriate use of each depending on the needs raised by the game at any time of its performance.

#### 3.2.2. Communication Layer

CAFCLA integrates different communication protocols to send and receive information between different physical devices: Wi-Fi, 4G/3G/GPRS and ZigBee to transmit data between mobile devices, from any sensor to the collectors and hubs, and to locate users. In addition, CAFCLA is an open framework that enables the integration of any other protocol that may be needed.

#### 3.2.3. Context-Awareness Layer

To gather contextual information, CAFCLA has integrated the wireless sensor network and the real time locating system platform:

- To deploy the wireless sensor network, CAFCLA includes n-Core [75] which uses the ZigBee communication protocol (IEEE 802.15.4). The sensors data are sent through the ZigBee network to data collectors, which, in turn, send the information collected to the server hosting the database through the Wi-Fi protocol. This technology allows for collecting the physical measures that permit the system to determine in real time the contextual status of the environment (temperature and luminosity sensors), instant and historical energy consumption of each job site (electricity consumption sensors), and the status of lighting and HVAC systems (on/off sensors). This information serves a threefold purpose: (i) it allows for knowing at all times the environmental and consumption parameters that draw the context; (ii) it provides real-time information to users; and (iii) it facilitates the analysis of the data and their use by other parts of the system.
- To provide the location, CAFCLA integrates n-Core Polaris [76], which allows for determining the position of users with up to one-meter accuracy based on the ZigBee wireless communication protocol. In order to locate, n-Core requires a set of beacons to be deployed, and they collect the signal sent by tags that are worn by the players. That signal, and its associated data, is sent to the server that implements the location engine, which calculates the position of each player. Players wear an n-Core Sirius Quantum tag responsible for sending the signal, and it is also equipped with an accelerometer that determines whether the user is moving. The beacons send these data to the server in the same way that sensor data are sent through Wi-Fi data collectors.

n-Core allows for the deployment of the wireless sensor network and the location system using the same platform and physical infrastructure. Furthermore, both systems share data collectors to send data to the server.

#### 3.2.4. Management Layer

This layer integrates the social machine, which is in charge of context-awareness and operation of the communication layers in a distributed, effective and predictable way. One of the biggest challenges that the development of Social Computing systems has to face is the communication and coordination between the participating entities, whether human or machine [23]. To address this challenge, we propose the use of virtual organizations of agents [77,78]. This technology dynamically creates agent organizations, defines functional behaviours such as schedules, tasks or services, and establishes logical structures and interactions, relationships or roles [20].

**Figure 2:**
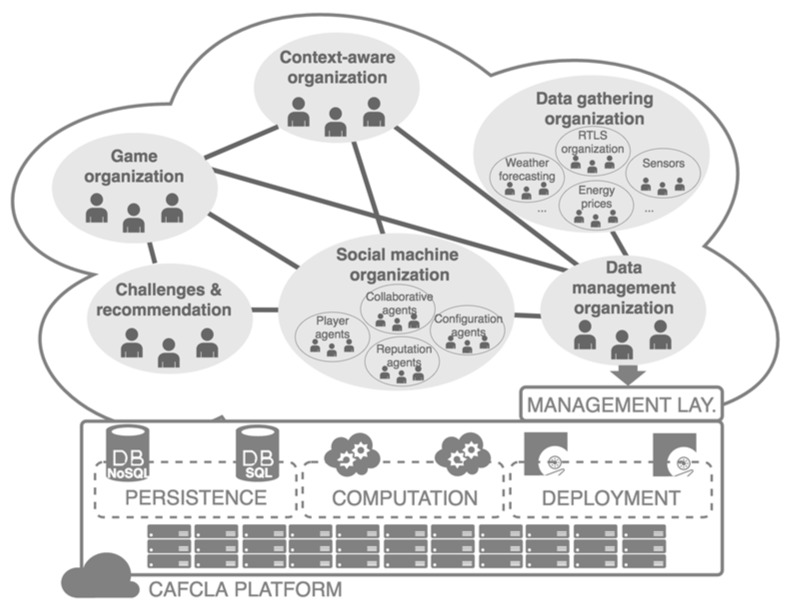
The virtual organization supports the social machine, which governs the recommendation of energy efficient actions and collective challenges.

The main purpose of the management layer is to implement the social machine using virtual organizations. The proposed architecture includes different organizations (see Figure 2):

- *Data gathering organization*: the data that are available to the system come from different sources that require a thorough control. This organization is responsible for managing these heterogeneous sources, such as sensor networks, the location system or even the published or consulted information, among others. Moreover, the organization is responsible of the reliability of the data collection, as well as the management of the security aspects related to this task.
- *Data management organization:* this organization is responsible for maintaining the integrity of data during the game. It makes decisions on what data should be elaborated and stored at all times. This organization classifies the information to be delivered, depending on the context and social information that surround the player at an instant. Moreover, the organization registers and stores the actions classified as energy efficient actions (actions that involved energy savings). Finally, this organization handles the security and integrity of all the data involved in the game.
- *Context-aware organization*: this organization manages the information collected by the sensor network. It needs to be coordinated with the data management organization to update the information from any physical service implemented by the sensor network.
- *Game organization*: the whole activity is under control of this organization (management and coordination). The information from the social machine (players, contextual data, information, etc.) is received and managed by this organization. It finally decides which information is provided to players according to the stage of the game.
- *Social machine organization*: this organization is responsible for performing analyses that extract socially relevant information related to the interaction of different agents:
  ⚬ *Player agents*: store the information related to the game process and are grouped in organizations, creating two types of interactions: player–player and player–machine.
  ⚬ *Configuration agent*: this agent creates, modifies and monitors the development of the game and establishes the social rules of the social machine organization.
  ⚬ *Collaborative agent*: it monitors the communication with the Context organization and the Activity organization. It is grouped in organizations.
  ⚬ *Reputation agent*: manages the reputation of actions. This reputation is based on the reliability (if they have involved energy savings) and the fidelity (if they have been continued over time). The social machine recommends the most reputed actions, where the reputation of action x during the day t is defined as:
    (1) $$Rep_{x,t}=\;{\bar{Fid}}_{x,t}*\;{\bar{Rel}}_{x,t},$$
    where ${\bar{Fid}}_{x,t}$ and ${\bar{Rel}}_{x,t}$ are the averaged fidelity and reliability of action x until the day t, respectively,
    $${\bar{Fid}}_{x,t}=\;\sqrt[{t}]{\left(1+Fid_{x,1}\right)\left(1+Fid_{x,2}\right)\ldots \left(1+Fid_{x,t}\right)}-1,$$
    $${\bar{Rel}}_{x,t}=\;\sqrt[{t}]{\left(1+Rel_{x,1}\right)\left(1+Rel_{x,2}\right)\ldots \left(1+Rel_{x,t}\right)}-1,$$
    and $Fid_{x,t}$ is the fidelity (i.e., the number of times the action x was performed during day t), and $Rel_{x,t}$ is the reliability (i.e., the net energy savings of action x during the day t).
- *Challenges and recommendations organization:* this organization is responsible for producing engaging personalized actions for the players to reach the objectives set in the Game organization.

The implementation of the virtual organization has been made through the PANGEA (Platform for the Automatic coNstruction of orGanizations of intElligent Agents) platform [79], a multi-agent architecture and organizational-based platform designed to facilitate the design and implementation of autonomous reactive and deliberative agents. The platform was developed to implement open multi-agent systems by providing several tools to create, manage and control virtual organizations, including organizational aspects. Its main features are: (i) the creation of organizations and sub-organizations; (ii) the management of roles; (iii) the management of services; (iv) the management of rules; (v) the management of security and (vi) the management of reliability of the system.

PANGEA permits the integration of agents developed in different languages, such as Java or C++. Moreover, the communication language used is IRC (Internet Relay Chat) although KQML (Knowledge Query and Manipulation Language) and FIPA-ACL (Foundation for Intelligent Physical Agents) are also integrated by the platform. In this paper, Java and IRC were the chosen languages to integrate the virtual organization of agents needed [79].

#### 3.2.5. Application Layer

The top layer in CAFCLA schema is the application layer. This layer supports the social and serious game development and provides the interface for players and game organizers, as well as other components that are a part of it, such as the configuration of different devices. As an implementation example, the next section presents the case study of a serious game where players were rewarded or penalized depending on their behaviour in energy savings within a work environment.

Furthermore, it is important to mention that CAFCLA provides several tools that establish collaboration between players (e.g., deciding on meeting times), and they can provide contextual information at any time, which is necessary so that players can make the best decisions, and it also gives recommendations on the decisions that could be taken in order to be rewarded (e.g., turn off the monitor when they finish working).

### 3.3. Trust and Security

Within the system developed, agents establish relationships with other agents, with other non-agent software or with humans. For this reason, the security and reliability during the game deployment is an important aspect to consider. The use of PANGEA, as the base platform for the creation of the virtual organizations of agents, permits the integration of security and reliability mechanisms in the game easily and intuitively [79].

**Figure 3:**
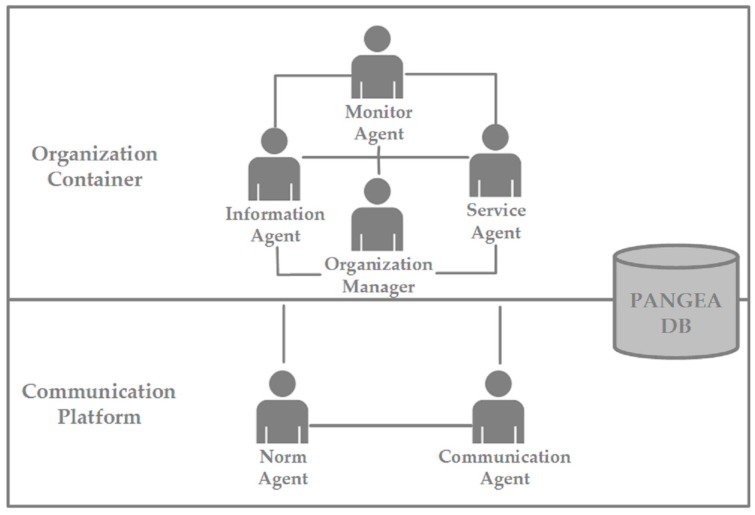
Overview of PANGEA (Platform for the Automatic coNstruction of orGanizations of intElligent Agents) platform architecture and the different agents that are implemented to manage virtual organizations and ensure their correct organization, security and reliability.

As can be seen in Figure 3, PANGEA provides a set of agents that permits the control of the organization in all aspects, including security and reliability. When developing the system presented in this paper, the integration of security and reliability has been organized in three different levels: data, operation and communication.

At the data level, the mechanisms deployed include verification of agent identity when accessing the data to ensure that it has the right permissions and that the appropriate information is delivered to it. The *Information Agent* is responsible of this task and manages the data access in the platform. Moreover, the *Organization Manager* establishes, coordinates and manages all permissions for an efficient data management.

On the other hand, at the operation level, multiple security and trust mechanisms are implemented. Among them, the registration of agents and the verification of their entrances and exits in the system. The *Organization Manager* controls both the registration and verification, and it is also in charge of the unique identification of the agents within the system and of the management of organizations and sub-organizations. Moreover, reliability is provided by the managing of the life cycle of the agents and by starting any agent in the case of failure. The *Monitor Agent* is in charge of these two important tasks in the system. Finally, to complete the security at operation level, the *Service Agent* records and controls the operation of all of the services.

Furthermore, the platform integrates security and reliability mechanisms at a communication level. In this sense, the *Norm Agent* ensures the compliance of all communications with the norms of the organization. All of the communications include encryption using TLS (*Transport Layer Security*) because of its easy integration in Java and IRC, and adopt standards defined by the use of languages such as IRC or KQML. Moreover, the *Communication Agent* controls the communication between agents and records the interactions among them to react properly in case of failure. Finally, the *Monitor Agent* is responsible for ensuring the secure communication between agents [79].

The following section presents and discusses the deployment and results obtained from the described serious game from the viewpoint of energy efficiency, noting the benefits of using CAFCLA and sensor networks for these types of games that are intended to promote a behavioural change.

## 4. Results and Discussion

In this section, we depict all of the issues related to the development of the game. Firstly, we briefly describe the serious game. Secondly, we give a description of the environment in terms of energy, the technical infrastructure deployed and the concrete energy efficiency measures that are taken during the game performance. After that, we present and analyse the obtained experimental results.

### 4.1. Serious Game Approach

The main objective of the serious game is to raise awareness in the efficient use of energy. Players were able to view the actions they have taken that have involved significant energy savings (turning off lights, not using HVAC, etc.). Since social computing was used, the game could make suggestions to the players on the most efficient actions that have been taken by others. These actions are identified due to the data collected by the sensors, the energy savings they entail and the probability that they will be maintained over time.

**Figure 4:**
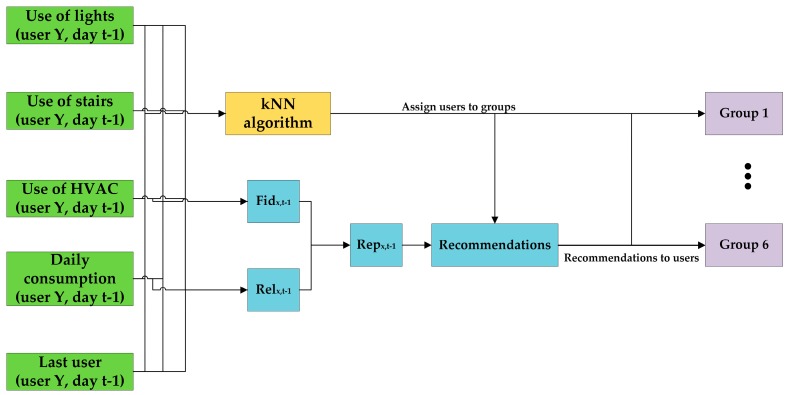
The social machine uses a kNN algorithm to classify the users into different groups based on their previous energy performance. In addition, it assigns a reputation to each action to be able to recommend them to the users who carry them out less.

The position of each user in the environment, combined with contextual information, determined the development of the game. All workers were involved in the game whose main objective was to get *virtual coins* through energy efficient behaviours. Each user received four recommendations per day in four different e-mails, determined and sent by the social machine, and they gave a clue on the action that should be taken. The users were clustered in six *energy saving groups* by means of the k-nearest neighbour (kNN) algorithm [80], considering the number of times they used the elevator, lighting and HVAC systems during the baseline period (see the next section and Figure 4). The social machine customizes all of the recommendations to each cluster according to the habits of the cluster (actions that users do not take into account will be recommended to foster a good habit) and to the reputation of the action (those that implied more energy savings got a better reputation than the ones with less energy saving impact).

To encourage participants, 250 virtual coins permitted players to grab a coffee or a soft drink for free. If he/she completed the action, he/she earned 10 virtual coins; otherwise, he/she was penalized with 10 virtual coins. Actions that helped win or lose virtual coins were as follows:

- Avoiding artificial lighting when natural lighting was greater than 200 Lux (200 Lux is the recommended luminosity for environments requiring moderate visibility lighting);
- Not using the HVAC system when it was above 18 °C in winter or below 25 °C in summer;
- Obtaining a daily electricity consumption below the average of the previous day;
- Using the stairs instead of the elevator;
- Turning off the lights and HVAC system when the last user left the laboratory;
- Belonging to the group that behaved more efficiently in a two-day period.

### 4.2. Sensoring Structure and Consumption Baseline

The proposed framework was assessed in one of the laboratories of the BISITE research group of the University of Salamanca. The selected dates for its performance tried to homogenize to the maximum the work conditions, as well as to minimize the influence of external factors. For these reasons, the beginning of the course, after summer holidays, was chosen, since, in this period, researchers have a more stable work load. In addition, it is important to mention that each of the participants performed the same functions and followed the same working schedule (working hours) during the two months of experimentation, so that the results were minimally influenced by external factors, changes in workload or holidays. Finally, to minimize the influence of weather conditions and sunshine hours: lower thermal oscillation among the months, similar sunlight hours during business hours and similar rainfall each month. According to all of these factors considered, the months of September and October were chosen to develop the game.

As can be seen in Figure 1, the lab has a common workspace, with an 88 square meter area, where the positions of the 18 people involved in the game are located. In addition, there are two separate meeting rooms, with 12 square meters each, which have enough space for eight people simultaneously. The laboratory is located on the second floor of the I+D+i (R&D&I) Building and can be accessed via an elevator or stairs.

Figure 1 shows how different points that measure temperature and luminosity were defined, consumption sensors were placed in each position, on/off sensors which determined the state of the lighting and HVAC system were located and the different areas where the game took place were defined (two meeting rooms, working area, 2nd and ground floor stairs and 2nd and ground floor lifts).

To monitor the power consumption a Cloogy [81] power plug was installed at each workstation, forming a total of 18. The plug includes an electrical consumption sensor, with an accuracy of ± 1% ± 0.5 W, and ZigBee communication capability. All of these sensors formed a ZigBee network through which real-time power consumption data for each position was transmitted. Consumption data was sent to the server every 15 min and in real time if the players asked for it. This data is collected by a crawler integrated within the Data Gathering Organization through a Web Service from the web page in which the consumption is published. With this data, users could check their electricity consumption at all times throughout the day, their consumption history and its comparison to the consumption of other players, which functioned as a motivational factor. Furthermore, these data allowed for determining which users were above and below the average consumption each day. It was intended that users were aware, for example, of the times that they should turn off their computers and monitors if they were not going to be used for long periods of time.

**Figure 5:**
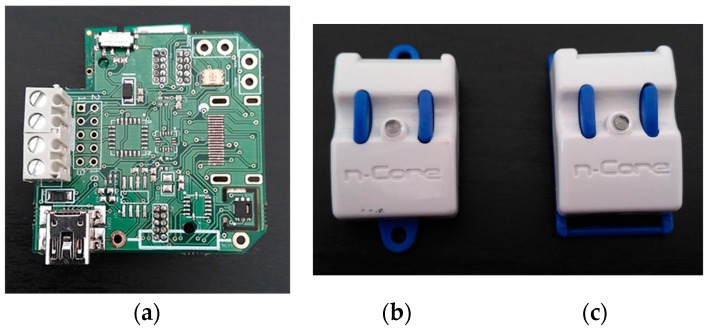
Sirius devices deployed to form the WSN and RTLS. (**a**) IOn-E sensor board, (**b**) Sirius RadIOn and (**c**) Sirius Quantum.

To encourage a more moderate use of HVCA and lighting systems, four IOn-E devices were deployed along the same wireless sensor network (see Figure 5). Two of these devices collected data in the shared work environment and one was located in each of the two meeting rooms. Each IOn-E device includes a SHT25 temperature sensor (Sensirion, Staefa, Switzerland), a TSL2561 light sensor (AMS, Stiria, Austria) and ZigBee communication capability by coupling it to a Sirius RadIOn device (Nebusens, Salamanca, Spain) through a digital I²C. SHT25 is a high-end band-gap temperature sensor that operates between –20 °C and 100 °C, with an accuracy of ± 0.1 °C when it is working between 0 °C and 60 °C, and works with 3 V VDD (Voltage Drain Drain). The TSL2561 light sensor also works with 3 V VDD, its dynamic range comprises from 0.1 to 40,000 Lux and automatically rejects 50/60-Hz lighting ripple. Each variation of 0.5 °C was sent and stored. Meanwhile, luminosity was sent to the server every 60 s. Moreover, temperature and luminosity could be asked by users under demand. Players knew at all times the data collected by these sensors so that they could assess whether the use of artificial lighting or HVAC systems was necessary or not, based on the premises set out in Section 3.2.5.

The wireless sensor network monitoring power consumption, temperature and luminosity also enabled the localization of all users within the work environment with an accuracy of 1 m and with a location period of 1 s. In addition, Sirius RadIOn devices were deployed (see Figure 5) both near the elevator and the stairs on the three floors of the building. Thus, the system could determine where the user is at any moment (stairs, elevator, meeting room, etc.). To locate the users, each one carried a Sirius Quantum device. As we can see in Figure 5, it works in the system as a location tag. This device sends different signal measurements (LQI (Link Quality Indication), RSSI (Received Signal Strength Indication)) every second through the ZigBee network to the server and its position in the system is calculated by the location engine. At the same time, the system could detect if the player was using of lighting or HVAC. Thus, the system was capable of determining if the user rose to the laboratory by elevator or using the stairs or which player was the last person leaving the workplace or a meeting room, recommending to turn off lights and air conditioning by using for this purpose the on/off sensors coupled in each lighting switch and HVAC thermostat.

The description of the environment, considering energy consumption, is focused on four different perspectives: lighting, HVAC, electrical energy consumption at all workplaces and use of the elevator. To obtain the baseline, the usual consumption without involvement in the game was monitored during one month. The results of this monitoring are as follows:

- *Laboratory lighting:* lightning enters naturally through the windows and a small courtyard. Furthermore, artificial light is provided by fluorescent lamps. The common working area is supplied with three lines of 58 W fluorescent tubes, while each meeting room is supplied with one line of these tubes. The lab is open from 08:00 a.m. to 09:00 p.m., 13 h a day during which lights are lit continuously in the work area. The energy consumption of the working area is 27.14 kWh every day. Similarly, the measure of the lighting consumption of each meeting room was taken before the start of the game during one month. The average data obtained indicates that the use of each room consumes 1.04 kWh per day. Total consumption of electricity for lighting in all units of the laboratory is 31.31 kWh per day.
- *A/C:* the air conditioning in the lab is given by the *HVAC system* of the building. However, each of the spaces has an individual thermostat that allows for turning the supply on and off and regulating the temperature in the area. This system is constantly in operation during the working hours and it is automatically disconnected when the building is closed. Before the game, none of the users took care of switching off the temperature either in the common area or in the meeting rooms.
- *Workstation*: each workstation is provided with an *LCD monitor and a laptop*. These two devices are fed through the same plug through which consumption is monitored by a Cloogy device. The measurement of consumption was made during the month before the game started, obtaining an average hourly consumption of 0.1535 kWh per player and 1.309 kWh per player per day. Moreover, it has been measured that the working hours of each site were 7.68. During the remaining hours, devices were on standby, obtaining an hourly stand by consumption of 0.018 kWh per player and 0.2937 kWh per player a day. With these data, the average hourly consumption measured for each player during one month was 0.1715 kWh.
- *Meeting rooms:* they were used by 15 users, who were organized in five meetings, lighting systems were used during all meetings and three of them made use of the HVAC system.
- *Elevator:* on the other hand, each user goes up and down to and from the second floor at least four times a day: when arriving to work in the morning, at break time, at lunch time and when leaving work. Lab workers were monitored on the use of the elevator: 12 of them use the elevator in almost 90% of cases either going up or down, while six of them always use the stairs.
- *Leaving lab:* last users leaving the lab did not turn off the lights or HVAC. Lights are not switched off at the end of the day in 80% of cases while the HVAC system always keeps working when everybody has left the laboratory.

### 4.3. Experimental Results

The game was developed following the guidelines outlined in the previous sections. The 18 workers of the BISITE research group participated in the game during 30 working days in their laboratory. The data obtained from the monitoring desktop showed that the average total consumption per day of all the users at their workstations was 2.875 kWh, and the hourly energy consumption per player at his workstation was 0.1597 kWh. These results established that there were savings of between 6.6% and 6.9% with respect to the measurements made before the game.

**Figure 6:**
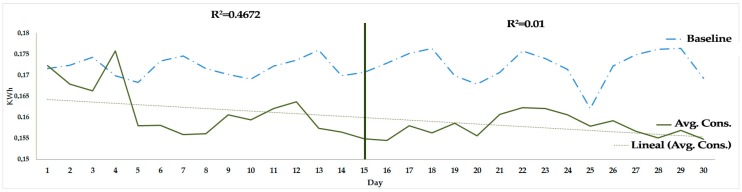
The graph of the hourly kWh average consumption of the desktop of each player provides evidence that there was a first phase in which players learned how to play the game and a second phase in which they maintained the good habits acquired, proving the effectiveness of the social machine.

Figure 6 shows the hourly kWh average consumption of each player’s desktop during the 30 days of the game evolvement. This graph provides evidence that there were two different phases during the development of the game (R^2^_1_ > R^2^_2_ where the independent variable is the number of days during the game development): during the first three weeks of development, the linear approximation of the average consumption set a value of R^2^_1_ = 0.47, in which players were more motivated and learned about the game; during the last three weeks of the game, the linear approximation of the average consumption set a value of R^2^_2_ = 0.01, indicating that the consumption remained more stable, always below the baseline of average consumption acquired in the data collection phase. The result indicates that, in the first phase, the players learned how to play the game, and, in the second phase, they maintained the good habits acquired, showing that the use of the social machine was effective.

Moreover, according to the hourly kWh baseline consumption per player, the use of the social machine in the game permitted an average of 7% energy savings per player and desktop. These savings were calculated as follows:

(2) $$Savings=\frac{Baseline\;consumption-Reported\;consumption}{Baseline\;consumption}.$$

Consumption is given in kWh when measuring electric consumption or in the number of times that energy-efficient actions were recommended and carried out.

On the other hand, we analyzed the energy consumption habits when using the meeting rooms. During the 30 days of game progress, it was observed that the use of artificial lighting was reduced by 56% and the use of the HVAC system by 82%. These savings were mostly through playing the game, an argument that is confirmed with the results of the performed Student’s *t*-test, reporting a mean of 2.15 times per day that lighting was used during the game, in comparison to the mean of 5.071 before the development of the game, and a *p-*value of 0.001, as can be seen in Table 2.

In addition, it was evident that all users were aware of turning off lights and thermostats if they were the last to leave the workplace, and this is largely due to the warning system integrated. During the 30 days period, both systems were turned off when the lab was closed except the first two days at the beginning of the game (see Table 2). These data indicate that the behavioural change promoted by the game was effective and significant; the mean and *p*-value values after performing Student’s *t*-test affirm this statement: the mean of the number of times per day that lights were not switched off when the last player left the laboratory decreased from 0.857 to 0.142, and reported a *p*-value of 0.000. Similarly, the mean of the number of times that HVAC was not switched off was reduced from 1 to 0.142, and reported a *p*-value of 0.000.

Finally, Table 2 shows that stairs were the option chosen in more than 88% of cases, as opposed to the 40% previously measured. In this case, a Student’s *t-*test was performed to prove the decrease in the number of times the lift was used; a mean of 4.933 was obtained for the number of times the elevator was used per day during the game, in comparison to the mean of 44.774 times before its development.

From the results, it is clear that the game is a powerful tool to create a habit whose outcome is huge energy savings and, in addition, healthy habits, thanks to the incentive to achieve better results in the game.

## 5. Conclusions

This paper presents a serious game based on the social computing paradigm that integrates advanced technologies through the CAFCLA framework, including wireless sensor networks and real-time locating systems. The inclusion of these technologies allows for having a contextual characterization of the environment as precisely as required, since they favor the integration of any type of sensors, actuators and, thanks to the RTLS, the position, tracking and activity monitoring of the players. In addition, the game integrates Virtual Organizations of agents to create a social machine that personalizes recommendations for users. This integration enables resolving human–machine interaction and context-awareness issues and achieves the main goal of the game: that users acquire good energy saving habits in public buildings, such as the work environment.

The game has been developed in one of the BISITE research group laboratories, compared to other similar games. We can asseverate that the use of the provided framework presents a great potential for the development of systems that are intended to promote a behavioural change in the energy consumption habits in users. The case study showed that social interactions foster growth of interest in improving individual performance through competition among players. Moreover, the acquisition of good energy habits was encouraged to benefit the group in places where awareness of energy consumption is often absent, such as the working environment. Finally, the simple game that has been developed has demonstrated the potential of the framework for the development of these kinds of solutions. This work has encouraged the authors to improve their work in progress by designing and developing more complex serious games in which their potential is exploited more comprehensively.

The implementation of the proposed systems will be easier to develop in the near future thanks to the increase of devices and gadgets with sensing capabilities available in the market. Technologies that provide context-awareness information, such as thermostats or lighting sensors, are becoming more accessible, including the indoor location systems. This allows for implementing the proposed solution with more functionalities, less difficulty of development and at affordable prices. Moreover, social networks and games over the Internet are becoming more popular. However, a relevant issue in this aspect will be the coordination between promoters, developers and technology manufacturers, guaranteeing data privacy and security, and getting good user engagement.

Moreover, after the experimentation, it has been detected that the behavioural change has been maintained by the users after the end of the game. Users have continued with good habits in the use of the elevator, lights and HVAC in the meeting rooms and when leaving the workplace. However, some aspects such as the optimization in the use of energy in the job site has not been maintained as expected, since the level of savings are lower to those that were obtained during the performance of the experimentation.

Finally, it is important to note that the flexibility of the CAFCLA framework is an added value in comparison to other solutions. This is because the integration of multiple technologies and communication protocols can substantially improve context-awareness, meeting the requirements of a large number of potential cases of use that could be implemented.

## Figures and Tables

**Table 1:** CAFCLA (Context-Aware Framework for Collaborative Learning Applications) layers diagram and technologies associated with each one.

Layer	Technologies
Physical	Tablet, smartphone, temperature, luminosity, on/off and consumption sensors, location beacons
Communication	Wi-Fi, ZigBee, 4G/3G/GPRS
Context-awareness	WSNs, Real Time Locating System
Management	Social Computing, Virtual Organization of Agents
Application	API, Web interface game

**Table 2:** Results of the Student’s *t*-test and Levene’s test performed to assess the difference of means and variances between the baseline usage data and the data collected during the game development. In all cases the percentage of use after the game is notably lower, with *p-*value always under 0.05, which shows that there is energy saving and behavioural change thanks to the game.

	Baseline		Game					
Variables	Mean	Stdr. Deviation	Mean	Stdr. Deviation	*t*	*p*-Value (2-Tailed)	F	*p*-Value
Meeting room light ^1^ (# times per day)	5.071	2.200	2.150	1.200	4.357	0.001	5.717	0.024
Meeting room HVAC ^2^ (# times per day)	3.143	1.703	0.559	0.565	5.386	0.000	10.944	0.003
Light when leaving ^3^ (# times per day)	0.857	0.363	0.142	0.363	4.298	0.000	0.934	0.343
HVAC when leaving ^4^ (# times per day)	1.000	0.000	0.142	0.363	11.787	0.000	12.480	0.002
Use of elevator ^5^ (# times per day)	44.774	2.355	4.933	8.035	17.803	0.000	4.598	0.042

^1^ Meeting room light: # times light is used while a meeting is taking place and illumination conditions are optimal; ^2^ Meeting room HVAC: # times HVAC is used while a meeting is taking place and temperature conditions are optimal; ^3^ Light when leaving: # times lights are not switched off when the last player leaves the laboratory; ^4^ HVAC when leaving: # times HVAC is not switched off when the last player leaves the laboratory; ^5^ Use of elevator: # times the elevator is used.
